# The *protein tyrosine phosphatase receptor type R *gene is an early and frequent target of silencing in human colorectal tumorigenesis

**DOI:** 10.1186/1476-4598-8-124

**Published:** 2009-12-16

**Authors:** Mirco Menigatti, Elisa Cattaneo, Jacob Sabates-Bellver, Valery V Ilinsky, Philip Went, Federico Buffoli, Victor E Marquez, Josef Jiricny, Giancarlo Marra

**Affiliations:** 1 Institute of Molecular Cancer Research, University of Zurich, Switzerland; 2 Department of Pathology, Triemli Hospital Zurich, Switzerland; 3 Gastroenterology Unit, Poliambulanza Hospital Brescia, Italy; 4 Laboratory of Medical Chemistry, National Cancer Institute, Frederick, MD, USA

## Abstract

**Background:**

Tumor development in the human colon is commonly accompanied by epigenetic changes, such as DNA methylation and chromatin modifications. These alterations result in significant, inheritable changes in gene expression that contribute to the selection of tumor cells with enhanced survival potential.

**Results:**

A recent high-throughput gene expression analysis conducted by our group identified numerous genes whose transcription was markedly diminished in colorectal tumors. One of these, the *protein-tyrosine phosphatase receptor type R *(*PTPRR*) gene, was dramatically downregulated from the earliest stages of cellular transformation. Here, we show that levels of both major *PTPRR *transcript variants are markedly decreased (compared with normal mucosal levels) in precancerous and cancerous colorectal tumors, as well in colorectal cancer cell lines. The expression of the *PTPRR-1 *isoform was inactivated in colorectal cancer cells as a result of *de novo *CpG island methylation and enrichment of transcription-repressive histone-tail marks, mainly H3K27me3. *De novo *methylation of the *PTPRR-1 *transcription start site was demonstrated in 29/36 (80%) colorectal adenomas, 42/44 (95%) colorectal adenocarcinomas, and 8/8 (100%) liver metastases associated with the latter tumors.

**Conclusions:**

Epigenetic downregulation of *PTPRR *seems to be an early alteration in colorectal cell transformation, which is maintained during the clonal selection associated with tumor progression. It may represent a preliminary step in the constitutive activation of the RAS/RAF/MAPK/ERK signalling, an effect that will later be consolidated by mutations in genes encoding key components of this pathway.

## Background

Epigenetic changes, such as aberrant DNA methylation and chromatin modifications, commonly accompany human tumor development (reviewed in [1,2]). They can have a dramatic impact on gene expression in tumor cells, and many contribute to the selection of cells with enhanced proliferation and survival potential. Indeed, as an evolutionary process, tumorigenesis derives enormous benefits from the plasticity implicit in epigenetic changes [3]. The large bowel is an excellent setting for the study of neoplastic progression, since colorectal lesions representing different stages of transformation can be collected and analyzed with relative ease. A recent high-throughput gene expression analysis conducted by our group identified numerous genes whose transcription is markedly diminished in precancerous and cancerous lesions of the gut [4], and many of these changes are likely to be the result of epigenetic alterations.

Several genes identified in our study were dramatically downregulated from the very earliest stages of cellular transformation. One of these was *PTPRR*, which encodes the classical transmembrane protein-tyrosine phosphatase (PTP) known as PTP, receptor type, R [5]. Reversible tyrosine-specific phosphorylation of cellular proteins is a key signalling mechanism used to evoke essential cell decisions such as proliferation and differentiation [6], and its proper regulation depends on the balanced activities of PTPs and protein tyrosine kinases (PTKs). Perturbed PTK signaling caused by mutations, amplifications, or chromosomal rearrangements results in deregulated kinase activity and malignant transformation [7]. Because they counteract PTK activity, PTPs were expected to have tumor-suppressive properties [8,9], and this view has been strengthened by data showing that members of the *PTP *superfamily are epigenetically silenced in several types of cancer [10-13]. Inactivating mutations of other *PTPs *have also been detected in several malignant tumors, particularly those of the colorectum [14]. On the other hand, certain PTPs have been shown to function as positive regulators of growth-stimulating signalling activated by cell-surface receptors, and gain-of-function mutations in the genes that encode these proteins have oncogenic effects [15].

In this study, we show that epigenetic silencing of the *PTPRR *gene is an early event in colorectal tumorigenesis. However, in addition to being detected in the vast majority of the precancerous lesions we examined, it was also present in almost all of the more advanced colorectal tumors, suggesting that downregulated *PTPRR *expression is also associated with clonal expansion. *PTPRR *silencing may thus represent a novel mechanism by which neoplastic colorectal cells evade tumor suppression.

## Results

**Figure 1:**
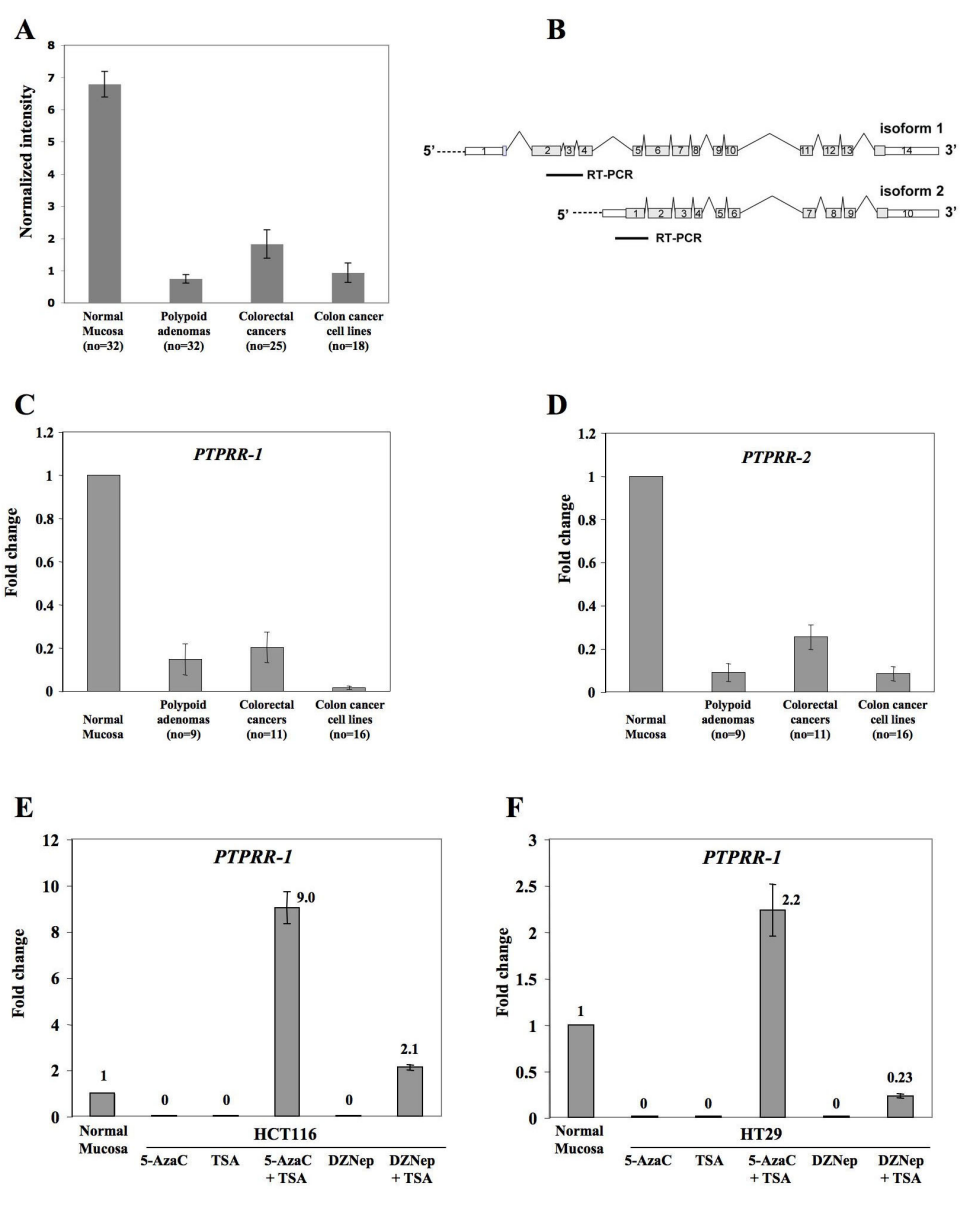
**Expression of *PTPRR *mRNA in normal colorectal mucosa, polypoid precancerous colorectal lesions, colorectal cancers, and colorectal cancer cell lines**. (**A**): mRNA levels based on normalized raw signal values (*y*-axis) were detected with the Affymetrix U133 Plus 2.0 arrays in the tissue sample series reported in Sabates-Bellver *et al*., 2007. (**B**): The two main isoforms of *PTPRR *are shown aligned from 5' to 3'. Introns have been reduced to minimal lengths, whereas exon sizes are proportional to their actual lengths. Horizontal bars represent the portions of cDNA that were amplified by qRT-PCR to discriminate between the two isoforms (see Methods). (**C**) and (**D**): *PTPRR-1 *and *PTPRR-2 *expression was investigated with real time qRT-PCR in a subset of the samples collected for the present study (see Methods). Fold changes were calculated as reported in Methods: For polypoid lesions and colorectal cancers, reference expression was the mean observed in the corresponding normal mucosa samples (indicated as 1); for cell lines, the reference consisted in the mean observed for 9 samples of normal mucosa from 9 patients with adenomas (indicated as 1). The differences between normal mucosa and the other groups of samples were highly significant in panels A, C, and D (all *p *values < 0.0001; bars: mean SE). (**E**) and (**F**): Two colorectal cancer cell lines (HCT116 and HT29) that do not express *PTPRR-1 *were used for the experiments shown in these panels. Re-activation of *PTPRR-1 *expression was investigated in cells treated with agents that act, through different mechanisms, on DNA and chromatin (see Methods). 5-Aza-dC, 5-Aza-2'-deoxycytidine; TSA, Trichostatin A; DZNep, 3-Deazaneplanocin A. Results are expressed relative to reference expression (mean observed in 9 different samples of normal mucosa, indicated as 1).

In our recent analysis of the transcriptomes of 32 colorectal adenomas [4], *PTPRR *emerged as one of the most markedly downregulated genes in these precancerous tissues. The same study also revealed dramatically reduced *PTPRR *transcript levels in 25 colorectal cancers and 18 colorectal cancer cell lines (Figure 1A), suggesting that *PTPRR *downregulation is an early but persistent alteration in colorectal carcinogenesis. In that study, we used the Affymetrix U133 Plus 2.0 array platform, which contains hybridization probes complementary to the 3' end of cDNAs. Consequently, it was impossible to discriminate between gene isoforms with differences limited to the 5' end, such as those described for *PTPRR *[16] (Figure 1B).

In the present study, we used real-time quantitative RT-PCR with specific primers to assess the levels of *PTPRR *transcript variants 1 and 2 (*PTPRR-1 *and *PTPRR-2*) in snap-frozen samples of colorectal tumors (precancerous and cancerous) and in colorectal cancer cell lines. As shown in Figures 1C and 1D, the expression of both isoforms was markedly decreased (compared with normal mucosa controls) in all 3 settings. (Table S1, Additional file 1 shows details for each sample.) Studies of their expression in other human normal tissues (Figure S1, Additional file 2) were consistent with previous reports [16]: The highest levels of the *PTPRR-1 *transcript were found in the brain, but the mucosae of the small intestine and colon were second and third on the list. As for *PTPRR-2*, the highest expression was found in the colon and then in the brain. In many of the other tissues examined, *PTPRR *expression was very low or absent, so its gene product seems to play fairly specific roles in the brain and lower gastrointestinal tract.

Our next goal was to determine whether the *PTPRR *downregulation we had observed was caused by epigenetic gene silencing. Colorectal cancer cell lines that do not express *PTPRR *were treated with the following agents - alone and in combination: 1) 3-deazaneplanocin A (DZNep), an S-adenosylhomocysteine hydrolase inhibitor that has been shown to selectively inhibit the trimethylation of lysine 27 on histone H3 (H3K27me3) and to reactivate silenced genes in cancer cells [17]; 2) the DNA methyltransferase inhibitor, 5-aza-2' deoxycytidine (5-AzaC); and 3) the histone deacetylase inhibitor, trichostatin A (TSA) (Figure 1E and 1F). While none of the treatments restored the expression of *PTPRR-2 *(data not shown), *PTPRR-1 *was re-expressed after combined treatment with 5-AzaC + TSA. Milder activation was achieved with DZNep + TSA. These data strongly suggest that *PTPRR *isoform 1 expression is being switched off in colorectal cancer cells as a result of *de novo *CpG methylation and other epigenetic mechanisms.

**Figure 2:**
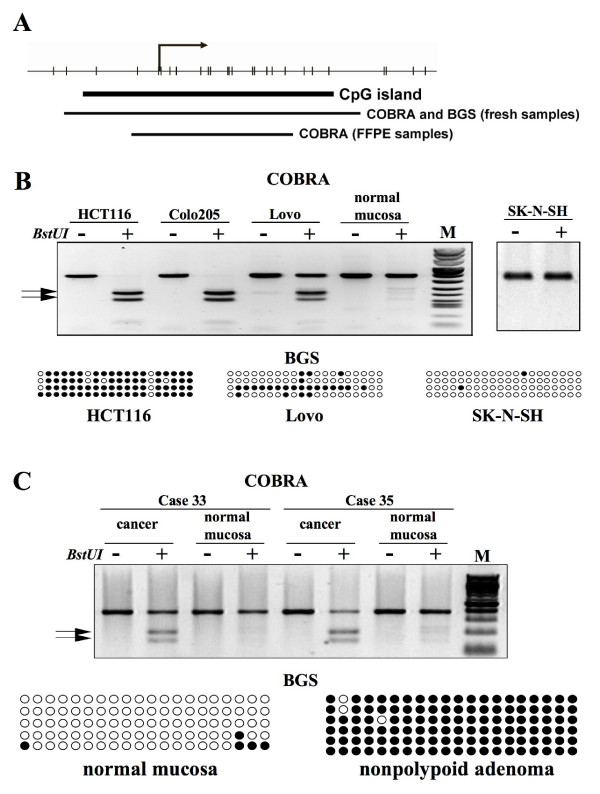
**Methylation analysis of *PTPRR-1***. (**A**): Schematic depiction of the *PTPRR-1 *CpG island encompassing the transcription start site (black arrow). CpG dinucleotides are represented as short vertical lines. Regions explored with combined bisulfite restriction analysis (COBRA) and with bisulfite genomic sequencing (BGS) on DNA extracted from fresh or formalin-fixed, paraffin-embedded (FFPE) samples are also shown. (**B**) and (**C**): Examples of COBRA analysis and BGS in cell lines (B) and clinical samples (C). COBRA: Arrows indicate *BstUI*-digested DNA fragments representing methylated alleles; slower-migrating fragments correspond to undigested, unmethylated DNA. BGS: Each row shows the methylation status of a cloned target sequence, with circles representing unmethylated (open) and methylated (filled) CpG dinucleotides.

We then used the EMBOSS CpGPlot program http://www.ebi.ac.uk/emboss/cpgplot/ to identify CpG islands in the genomic DNA regions around the transcription start sites (from -10 kb to +0.5 kb) of the two isoforms. Results were negative for the *PTPRR-2 *start site, but that of *PTPRR-1 *was surrounded by a 228-bp CpG island (Figure 2A). Combined bisulfite restriction analysis (COBRA) was used to explore the methylation status of this genomic area in the 16 colorectal cancer cell lines listed in Supplementary Table 1 and in the glioblastoma cell lines SK-N-SH, SK-N-AS, and LAN1, all 3 of which express the *PTPRR-1 *transcript (data not shown). As expected, the CpG island showed no sign of methylation in any of the glioblastoma lines, but it was partially methylated in Lovo cells and extensively methylated in the other 15 colorectal cancer cell lines (Figure 2B and Supplementary Table 1). The specificity of these results was confirmed by sequencing of subcloned PCR products of bisulfite-converted DNA from HCT116, Lovo, and SK-N-SH cells (Figure 2B). COBRA also revealed aberrant methylation in this CpG island in the vast majority of precancerous and cancerous tissues we tested and in all 8 liver metastases from colorectal cancers (Table 1 and Figure 2C). For 21 of the lesions included in this analysis (5 adenomas --3 nonpolypoid, 2 polypoid-- and 16 cancers), corresponding samples of normal mucosa were also available for methylation analysis. None of these samples met our pre-established definition of significant methylation (i.e., involvement of ≥ 5% of alleles -- see Materials and Methods), but 11 presented very low-level methylation suggestive of a possible field defect.

**Table 1:** Methylation in the CpG island encompassing the *PTPRR *isoform 1 start site in colorectal tumors.

Tumor type^1^	No.	Methylated (%)^2^	Unmethylated (%)
**Premalignant lesions**			
Polypoid	28	23 (82)	5 (18)
Nonpolypoid	18	15 (83)	3 (17)
**Primary adenocarcinomas**	44	42 (95)	2 (5)
**Liver metastases^3^**	8	8 (100)	0 (0)

^**1 **^The normal mucosa counterpart was also analyzed for 21 samples (see Results)

^**2 **^Positivity threshold: ≥ 5% alleles (see Methods)

^**3 **^Associated with 8 of the 44 primary cancers listed above

**Figure 3:**
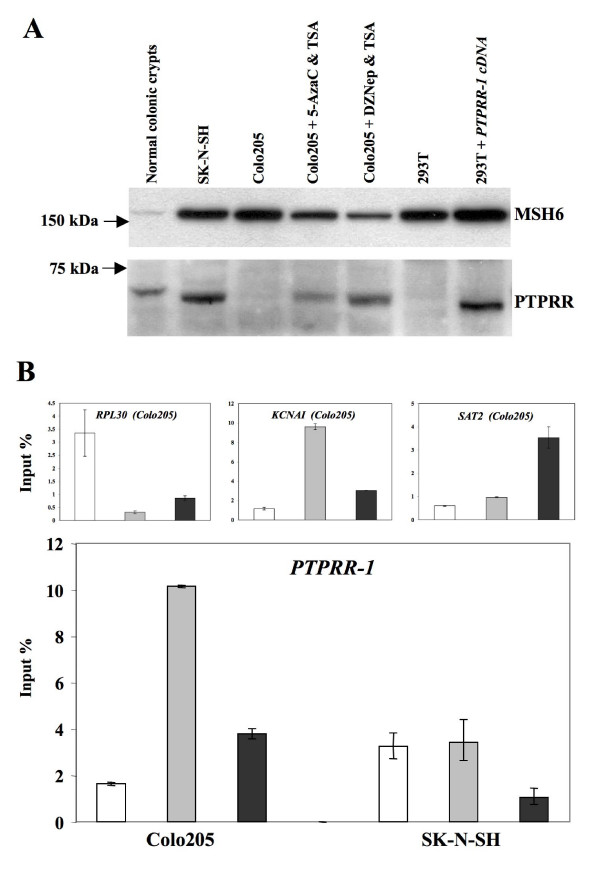
**Western blotting and chromatin immunoprecipitation (ChIP) studies**. (**A**): PTPRR-1 protein expression in total cell extracts from the cell lines used for ChIP studies (Colo205 and SK-N-SH) are shown. Proteins were separated on a 10% polyacrylamide gel. Reactivation of PTPRR protein expression was also investigated in Colo205 cells after treatment with 5-Aza-2'-deoxycytidine (5-Aza-dC) or 3-Deazaneplanocin A (DZNep) plus Trichostatin A (TSA). Extracts of 293T cells transiently transfected with *PTPRR-1 *cDNA were used as positive controls. PTPRR expression was also documented in assays of protein extracts from epithelial cells of normal colonic crypts, as expected. (The slightly higher molecular weight might be due to a post-translational modification occurring in vivo in this tissue.) MSH6 antibodies were used as proliferation-rate controls: In cells treated with drugs, the expression of the DNA mismatch repair protein MSH6 was decreased --- as expected --- due to reduced cell viability. (**B**): Immunoprecipitated DNA was quantified by real-time PCR. Enrichment was calculated as percentage of input DNA control (details in Methods). Upper 3 panels: Chromatin prepared from Colo205 cells was immunoprecipitated with antibodies against H3K9ac (acetylated-histone H3Lys9, white bars), H3K27me3 (trimethyl-histone H3Lys27, light gray bars), and H3K9me3 (trimethyl-histone H3Lys9, dark gray bars). The specifity of immunoprecipitations was verified by the selective enrichment, in the PCR amplifications, of the actively transcribed *RPL30 *gene (H3K9ac antibody), the polycomb target *KCNA1 *promoter (H3K27me3 antibody), and the heterochromatic *Sat 2 *locus (H3K9me3 antibody). Lower panel: *PTPRR-1 *promoter histone code differences between the colorectal cancer cell line Colo205 and the neuroblastoma cell line SK-N-SH. (*PTPRR-1 *transcript and PTPRR-1 protein expression profiles for the 2 lines are shown in Figures 2B and 3A, respectively.)

The fact that *PTPRR-1 *expression was reactivated in colorectal cancer cell lines by treatment with 5-AzaC + TSA or DZNep + TSA strongly suggested that the tumor-associated silencing of this transcript might be due not only to CpG island hypermethylation but also to histone code changes. Therefore, we used chromatin immunoprecipitation to investigate chromatin marks in the region around the *PTPRR-1 *transcription start site (-28 to +156 bp) in SK-N-SH glioblastoma cells, which are known to express *PTPRR-1 *transcript and protein, and Colo205 colorectal cancer cells, which express neither (Figure 3A). Compared with SK-N-SH cells, the Colo205 line presented enrichment of the transcription-repressive histone-tail marks H3K9me3 and H3K27me3 and depletion of the active mark H3K9ac (Figure 3B). This histone code profile in Colo205 cells was almost identical to that of the *KCNA1 *gene, a well-known target of the transcription-repressing polycomb-group proteins in colorectal carcinogenesis [18]. Our findings thus suggest that the *de novo *methylation of the *PTPRR-1 *we document here may also be polycomb-mediated.

## Discussion

The results presented above demonstrate for the first time that human colorectal cell transformation is associated with silencing of both of the principal transcript variants of the *PTPRR *gene. More specifically, precancerous tumors and cancers of the colorectum are characterized by marked underexpression of *PTPRR-1 *and *PTPRR-2 *(also known as *PTPPBSα *and *PTPPBSγ*) [16,19]. Although the 2 isoforms are concomitantly downregulated in most tumors, the mechanisms underlying these effects appear to be different.

Silencing of *PTPRR-1 *was associated with *de novo *methylation of a CpG island encompassing its transcription start site. None of the normal colorectal tissues we examined displayed significant methylation at this site, but it was very common in adenomatous polyps (80%) and adenocarcinomas (95%), and also in liver metastases associated with the latter tumors (100%). This epigenetic phenomenon thus appears to be an early alteration in the transformation process that is maintained during the clonal selection associated with tumor progression.

The fact that it was also associated with H3K27 trimethylation was of particular interest. This transcription-repressive chromatin modification is catalyzed by EZH2, a component of the polycomb repressive complex 2 [20], and it plays key roles in developmental processes such as X chromosome inactivation [21], imprinting [22], and stem cell maintenance [23]. More recent research has shown, however, that polycomb-regulated genes are also frequent targets for *de novo *CpG-island methylation in cancer cells [24], a process triggered by the cancer-cell-specific recruitment of DNA methyltransferases by the polycomb repressive complex 2 itself [25]. These findings strongly suggest that the epigenetic silencing of *PTPRR-1 *is polycomb-mediated, and this hypothesis is strengthened by the fact that the expression of this isoform in colorectal cancer cell lines was reactivated by the histone methylation inhibitor DZNep, although the effect was seen only when this agent was combined with the histone deacetylase inhibitor TSA. DZNep alone is sufficient to reactivate the transcription of polycomb-repressed genes without methylated CpG islands [17,26], but the presence of CpG methylation leads to the addition of a second transcription-repressing histone mark, H3 *deacetylation*, through a process that involves selective binding of methyl-CpG-binding proteins [27,28]. For this reason, elimination of the epigenetic block affecting *PTPRR-1 *also required the action of TSA.

As for *PTPRR-2*, the mechanism underlying its silencing is still obscure. Although its tumor-related downregulation is almost always associated with that of *PTPRR-1 *(Supplementary Table 1), its transcription start site is located more than 150 kb downstream from that of the latter isoform, and its 5' flanking region --- unlike that of *PTPRR-1 *--- is CpG-poor. Consequently, the fact that *PTPRR-2 *was not re-expressed in the colorectal cancer cell lines treated with 5-AzaC + TSA was not surprising. However, treatment with DZNep + TSA also failed to reactivate the expression of this isoform, which tends to exclude the involvement of polycomb proteins in the silencing of *PTPRR-2 *expression in colorectal tumors.

The PTPRR-1 protein is a receptor-type protein tyrosine phosphatase, whereas PTPRR-2 is a shorter isoform found in the cytosol [16,19]. However, both contain a kinase-interaction motif (KIM) and a protein tyrosine phosphatase (PTP) domain [29]. The roles of the 2 PTPRR isoforms in humans are still unclear, but the functions of their homologs in mice and rats have been investigated more extensively. *In vitro *studies have shown that the KIMs of these proteins interact with various MAP kinases, including several extracellular regulated kinases (ERKs). PTPRR-mediated dephosphorylation of ERKs 1, 2, and 5 prevents their translocation to the nucleus [30-33]. The roles of these kinases as physiologically relevant PTPRR substrateshave also emerged from *in vivo *studies, which revealed significantly increased levels of phosphorylated ERK1/2 in the brains of *Ptprr*-knock-out mice [34]. Signalling through the ERK1/2 pathway is known to contribute to the survival of colorectal tumor cells (reviewed in [35]), and PTPRR is expressed in the normal colorectal epithelium [16] and Figures 1 and 3]. Therefore, although the *Ptprr*-/- animals cited above did not display any evident predisposition toward tumor development (34), a different picture might emerge in a setting of multiple gene changes. It would be interesting, in fact, to see how Ptprr deficiency affects the onset and/or progression of intestinal tumors in *Apc*^Min/+ ^or *KRAS*^*V*12*G*^/*Apc*^+/1638*N *^mice, which are used to study the role of concomitant gene alterations in colorectal tumorigenesis [36-38].

Our assessment of *PTPRR *expression levels in different human tissues (Supplementary Figure 1) reveals preferential expression in the brain and lower gastrointestinal tract, so its downregulation might reasonably be expected to lead to the development of disease in these tissues. The *Ptprr*^-/- ^deficient mice mentioned above [34] were viable and fertile, but displayed notable defects in fine motor coordination and balance skills. Future studies should also examine possible links between loss or reduced expression of PTPRR in the nervous system and neurological and psychiatric disorders.

Further investigation is also needed to determine the functional relevance of the *PTPRR *downregulation we documented in colorectal tumors, but one interesting possibility is that this change represents a mechanism for early establishment in colon tumor cells of "epigenetic sensitization" to the activation of oncogenic signalling (reviewed in [39,40]), in this case, through the RAS/RAF/MAPK/ERK pathway. This phenomenon has been proposed for genes with gatekeeper functions, which are silenced during early stages of the neoplastic process. A classic example is the early epigenetic loss of secreted frizzled-related proteins (*SFRP*), which compete with secreted Wnt proteins for binding to their receptor Frizzled. The constitutive Wnt signalling unleashed by this epigenetic event [41] is believed to precede genetic alterations, i.e., mutations, in key components of the pathway, such as the *APC *gene, whose inactivation is traditionally considered the trigger of colorectal tumor progression. Aberrant signalling through the Wnt pathway is largely responsible for expansion of stem-cell and progenitor-cell populations normally confined to the lower portions of intestinal crypts. The importance of the epigenetic contribution to Wnt pathway activation is difficult to determine with precision, but the possibility has been raised that it is essential for promoting tumor development or progression [39].

Epigenetic silencing of *PTPRR *might play a similar role in aberrant activation of the RAS/RAF/MAPK/ERK pathway. Like canonical Wnt signaling [42], MAPK activity has been shown to be restricted to the nuclei of proliferative, undifferentiated cells in the lower portion of normal intestinal crypts [43]. Constitutive RAS/RAF/MAPK/ERK pathway activity has been demonstrated in numerous primary human tumors of the colon, kidney, and lungs [44]. It is generally associated with gain-of-function mutations in the *KRAS *or *BRAF *gene [45,46], which are frequent in colorectal transformation [47]. Epigenetic downregulation of *PTPRR *might represent a very early step toward complete activation of the RAS/RAF/MAPK/ERK signalling, an effect that will later be consolidated by the addition of activating mutations in genes encoding key factors in this pathway.

## Conclusions

Our analysis of a large series of human tumors and cell lines indicates that epigenetic downregulation of *PTPRR *occurs early in colorectal cell transformation and is maintained during the clonal selection associated with tumor progression. Better understanding of the functional effects of PTPRR loss could shed light on important aspects of the mechanisms underlying colorectal tumor cell survival, in particular on the dynamics of constitutive activation of the RAS/RAF/MAPK/ERK signaling. The high frequency of *PTPRR *promoter hypermethylation in precancerous colorectal lesions could also have important implications for current efforts to develop stool- or serum-based DNA assays for early noninvasive detection of colorectal neoplasia.

## Materials and methods

### Cell lines and tissue samples

Colorectal cancer (Vaco481, Colo205, SW48, LS174T, SW620, HT29, Caco2, Colo741, HCT116, LS411, Lovo, SW480, SW837, GP5D, CX1, and CO115) and neuroblastoma (SK-N-SH, SK-N-AS, LAN1) cell lines were obtained from the Zurich Cancer Network's Cell Line Repository. Cells from this repository have undergone only a few passages since purchase (from the American Tissue Culture Collection, Teddington, UK), and are certified to be free from mycoplasma infection.

Fresh-frozen or formalin-fixed, paraffin-embedded neoplastic tissues (polypoid [n = 28] and nonpolypoid [n = 18] colorectal adenomas, advanced colorectal cancers [n = 44], and liver metastases related to 8 of the 44 cancers) were collected at the Triemli Hospital in Zurich, Switzerland and the Poliambulanza Hospital in Brescia, Italy with institutional review board approval. Twenty-one of the neoplastic tissue samples (3 polypoid adenomas, 2 nonpolypoid adenomas, 16 cancers) were accompanied by samples of normal mucosa from the same colon segment (≥ 10 cm from the lesion). The analysis of *PTPRR *transcript variant expression (Figure 1C and 1D) also included 6 other polyp-normal mucosa sets reported in our previous study (1). Genomic DNA was extracted with the QIAamp DNA Mini Kit (Qiagen, Basel, Switzerland). Total RNA was extracted from homogenized frozen tissue samples and cell lines with the RNeasy Mini kit (Qiagen).

### Drug treatment of cell lines

HCT116, HT29, and Colo205 cells were treated the day after seeding. For DNA demethylation, cells were grown for 72 hours in medium containing 5 μM 5-Aza-dC (Sigma, Buchs SG, Switzerland) (renewed every 24 hrs). In some experiments, these cells were then transferred to medium containing TSA (final concentration 300 nM) (Sigma) for an additional 16 hours' growth. To inhibit histone methylation, the same 3 cell lines were treated for 48 h with 5 μM of the S-adenosylhomocysteine hydrolase inhibitor 3-deazaneplanocin A (DZNep, provided by the National Cancer Institute, USA). Some DZNep-treated cells were then transferred to medium containing 200 nM of TSA for an additional 24 hours' growth.

### Real-Time Quantitative Reverse-Transcription Polymerase Chain Reaction (qRT-PCR)

First-strand cDNA synthesis was performed with the Transcriptor First Strand cDNA Synthesis kit (Roche, Basel, Switzerland) according to the manufacturer's instructions. Expression of *PTPRR *(GeneBank, GeneID:5801) isoforms and of the reference housekeeping gene *porphobilinogen deaminase *(*PBGD*, GeneBank, GeneID: 3145) was measured with the Roche LightCycler 480 Real-Time PCR System and a LightCycler 480 SYBR Green I Master kit. In accordance with the Pfaffl method [48], relative *PTPRR *isoform expression in 2 different tissue samples (e.g., a colorectal cancer versus corresponding normal mucosa) was based on the mean crossing-point (Cp) deviation between the 2 samples normalized to the mean Cp deviation for the reference gene, after efficiency correction of the PCR reactions. All primer sequences and PCR conditions are reported in Table S2, Additional file 3.

### Bisulfite conversion, combined bisulfite restriction analysis (COBRA), and bisulfite sequencing

Sodium bisulfite conversion of genomic DNA was performed as previously described [49]. COBRA was used to determine the methylation status of the CpG island encompassing the *PTPRR-1 *start site in bisulfite-modified DNA. Amplifications were carried out with FastStart Taq DNA Polymerase (Roche). The amplified products were digested with the *BstUI *restriction enzyme (New England Biolabs, Beverly, MA, USA) and subjected to 2% agarose gel electrophoresis and ethidium bromide staining. The methylation level (%) was calculated by dividing the sum of the densities of the shifted bands by the sum of the densities of all bands in each lane and multiplying the quotient by 100. Both sums were computed automatically by the ImageQuant software (Molecular Dynamics, GE Healthcare, Piscataway, NJ, USA). Samples with methylation levels < 5% were defined as methylation-negative.

For sequencing of bisulfite-converted DNA, PCR products were digested with *Eco*RI and *Bam*HI (New England Biolabs) and subcloned into the pGEM-7Zf(+) Vector (Promega, Madison, WI, USA). Individual clones were then sequenced with the PRISM dye terminator cycle sequencing method (Applied Biosystems, Rotkreuz, Switzerland).

### Chromatin immunoprecipitation (ChIP) assays

Experiments were performed with the ChIP-IT kit from Active Motif (Carlsbad, CA, USA) according to manufacturer's instructions. Colo205 and SK-N-SH cells were cross-linked with 0.8% formaldehyde at room temperature for 6 min, and glycine (0.125 M) was added to stop the reaction. Isolated nuclei were then subjected to 10 cycles (30 s on/30 s off) of sonication at 200 W with a Bioruptor sonicator (Diagenode, Liege, Belgium). Ten percent of the sonicated chromatin was reserved for use as an input DNA control. Sodium butyrate (Sigma) was added to cell-lysis and chromatin-shearing buffers (final concentration, 5 mM) to prevent endogenous histone deacetylase activity during the procedure. DNA-protein complexes were immunoprecipitated with antibodies against trimethyl-histone H3(Lys9) (ab8898, 1:100 dilution), acetylated-histone H3(Lys9) (ab4441, 1:100 dilution) - both from Abcam (Cambridge, UK), or trimethyl-histone H3(Lys27) (antibody 39535, 1:100 dilution; Active Motif).

The amount of immunoprecipitated target was measured with real-time PCR (Roche LightCycler 480 Real-Time PCR System and Qiagen's QuantiTect SYBR Green kit) and expressed as a fraction of input DNA (arbitrary value, 100). Table S2, Additional file 2 lists the primer sequences for amplicons located in the *RPL30 *gene, in the *KCNA1 *gene promoter, in the *Sat 2 *locus, and in the 5' region flanking the transcription start site of the *PTPRR-1*.

### Western blotting

RIPA buffer (25 mM Tris-HCl pH 7.6; 150 mM NaCl; 1% NP-40; 1% sodium deoxycholate; 0.1% SDS, 1× Roche COMPLETE mini protease inhibitor, and 1 mM PMSF) was used to extract total protein from SK-N-SH and Colo205 cells (treated or untreated), 293T cells, and 293T cells transiently transfected with with pcDNA3 vector carrying full-length *PTPRR-1 *cDNA. A previously described protocol [50] was used to obtain a total protein extract from epithelial crypt cells isolated from a specimen of normal colon immediately after surgical excision for diverticulitis. Western blotting was performed as previously described [51] using primary monoclonal antibodies directed against PTPRR (B01P, 1:1000 dilution, Abnova, Taipei City, Taiwan;) or MSH6 (G70220, 1:2000 dilution, BD Transduction Laboratories, San Jose, CA, USA). Abnova B01P was the only commercially available anti-PTPRR antibody that performed well in our Western blotting assays, but none of these products performed satisfactorily when we attempted to stain tissue samples (data not shown).

## Abbreviations

(PTPRR): protein tyrosine phosphatase receptor type R; (5-AzaC): 5-aza-2' deoxycytidine; (TSA): trichostatin A; (DZNep): 3-deazaneplanocin A; (COBRA): combined bisulfite restriction analysis; (BGS): bisulfite genomic sequencing; (qRT-PCR): real-time quantitative reverse-transcription polymerase chain reaction; (ChIP): chromatin immunoprecipitation.

## Competing interests

The authors declare that they have no competing interests.

## Authors' contributions

MM prepared the manuscript and performed methylation and chromatin studies; EC was responsible for the extraction of nucleic acids; JS-B performed the microarray analyses; VVI carried out the studies on cell lines, including protein extraction; PW analyzed and histologically classified tumor samples; FB performed the endoscopies; VIM and JJ designed important experiments during the study and contributed to the writing of the manuscript; GM conceived the project and prepared the manuscript. All authors have read and approved the final manuscript.

## Supplementary Material

Additional file 1 **Supplementary Table 1**. Expression of PTPRR transcript variants 1 and 2 in colorectal tumors and colorectal cancer cell lines as measured with real time quantitative RT-PCR. The expression of both isoforms in 9 polypoid adenomas, 11 colorectal cancers, and 16 colon cancer cell lines is shown. Click here for file

Additional file 2 **Supplementary Figure 1**. Expression of the two PTPRR transcript variants in a series of normal human tissues. The expression of the two PTPRR isoforms in different normal human tissues as measured with real time quantitative RT-PCR is shown. Click here for file

Additional file 3 **Supplementary Table 2**. Primer sequences and PCR conditions used in this study. All primer sequences and PCR conditions used for quantitative RT-PCR, methylation analysis, and ChIP are reported. Click here for file
