# Supplementary material for: The protein tyrosine phosphatase receptor type R gene is an early and frequent target of silencing in human colorectal tumorigenesis
Source: Mol Cancer. 2009 Dec 16;8:124. doi: 10.1186/1476-4598-8-124 (PMC2801661; doi:10.1186/1476-4598-8-124)
Supplement: Additional file 1 — Supplementary Table 1. Expression of PTPRR transcript variants 1 and 2 in colorectal tumors and colorectal cancer cell lines as measured with real time quantitative RT-PCR. The expression of both isoforms in 9 polypoid adenomas, 11 colorectal cancers, and 16 colon cancer cell lines is shown. [file 1476-4598-8-124-S1.PDF]

Supplementary Table 1: Expression of *PTPRR* transcript variants 1 and 2 in colorectal tumors and colorectal cancer cell lines as measured with real time quantitative RT-PCR.

	FOLD CHANGES[1]	
	PTPRR-1	***PTPRR-2***
**POLYPOID ADENOMAS[2]**		
AD-A4*	0.0002	0.048
AD-A26	**0.3548**	0.416
AD-A36	**0.6238**	0.067
AD-G5A*	0.037	0.014
AD-G7A	0	0.0152
AD-A27*	0.0004	0.058
AD-Vi.po. 16*	0.025	0.092
AD-Vi.po. 11*	0.0804	0.037
AD-A31*	0.1911	0.057
**COLORECTAL CANCERS**		
CRC-G9K	0.272	0.206
CRC-G13K	0	0.104
CRC-G15K	0.249	0.445
CRC-G17K	0.538	0.265
CRC-G23K	0.324	0.179
CRC-G25K	0.045	0.3
CRC-G27K	0.0017	0.015
CRC-G29K	0.045	0.369
CRC-G31K	0	0.083
CRC-G35K	0.08	0.688
CRC-G37K	0.675	0.139
**COLON CANCER CELL LINES**		
Vaco481	0	0.102
Colo205	0	0
SW48	0	0
LS174T	0	0.195
SW620	0	0
HT29	0	0.041
Caco2	0.071	0.12
Colo741	0	0
HCT116	0	0
LS411	0.059	0.118
Lovo	0.105	0.415
SW837	0.057	0
GP5D	0	0
SW480	0	0
CX1	0	0
CO115	0	0.347

[1] Fold changes were calculated as reported in Methods. The level in each tumor was compared with that found in normal mucosa from the same patient; levels in cell lines were compared with a single control level representing the mean found in 9 normal mucosal samples from patients with adenomas. Samples whose values are shown in boldface were found unmethylated by COBRA (Table 1). Each sample was analyzed in duplicate.

[2] Adenomas marked with asterisks (and corresponding samples of normal mucosa) were collected for a previous study (Sabates-Bellver *et al.*, 2007). All other tumor samples listed in the Table are described in the Methods section.
