# Supplementary material for: The protein tyrosine phosphatase receptor type R gene is an early and frequent target of silencing in human colorectal tumorigenesis
Source: Mol Cancer. 2009 Dec 16;8:124. doi: 10.1186/1476-4598-8-124 (PMC2801661; doi:10.1186/1476-4598-8-124)
Supplement: Additional file 2 — Supplementary Figure 1. Expression of the two PTPRR transcript variants in a series of normal human tissues. The expression of the two PTPRR isoforms in different normal human tissues as measured with real time quantitative RT-PCR is shown. [file 1476-4598-8-124-S2.PDF]

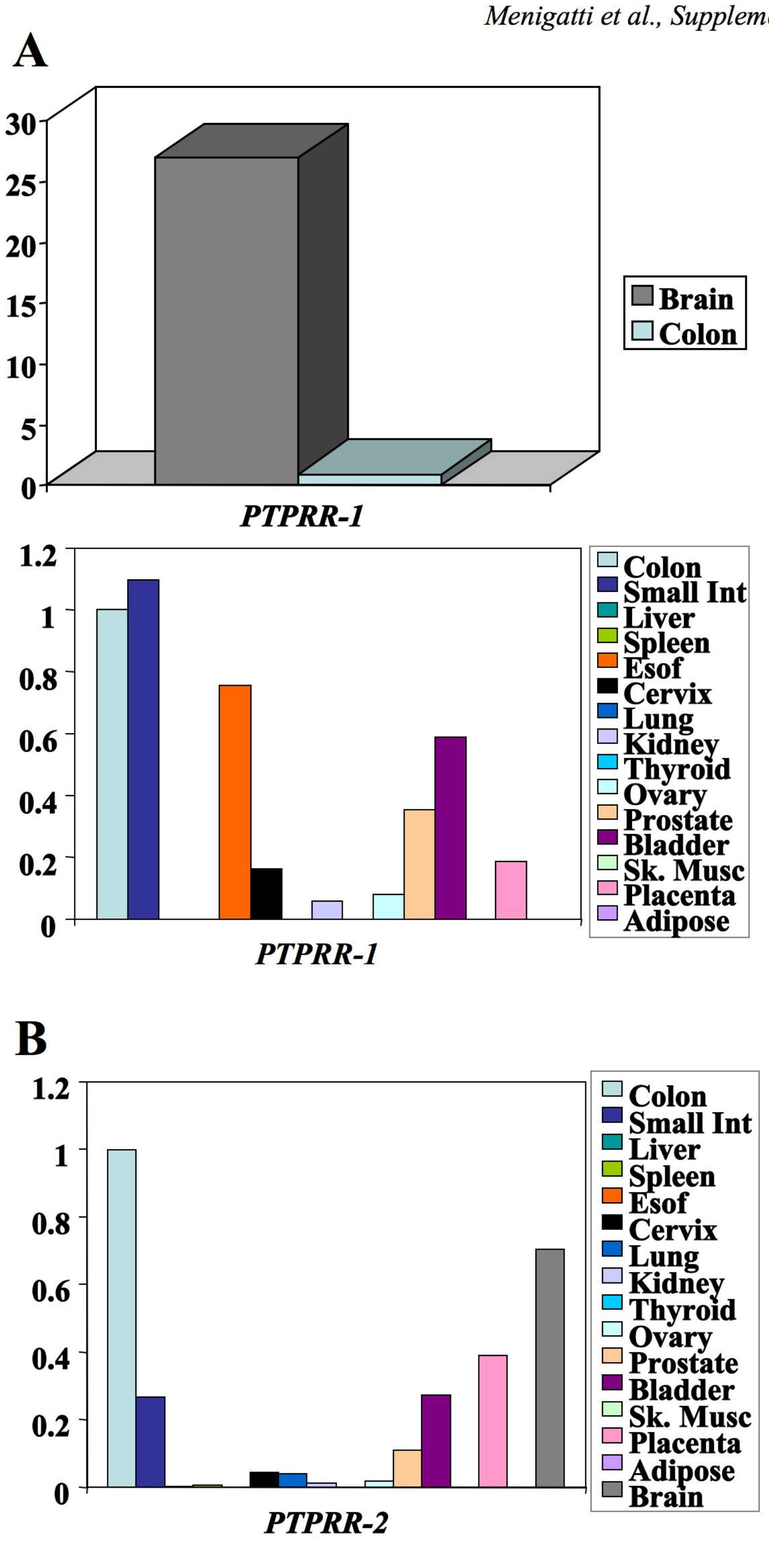

Supplementary Figure 1: Expression of the two *PTPRR* transcript variants (*PTPRR-1* in panel A, and *PTPRR-2* in panel B) in a series of normal human tissues. Real time quantitative RT-PCR was performed as described in Methods. Fold changes in the expression of the two transcripts were normalized to those detected in the normal colorectal mucosa sample indicated as level 1. Total RNAs extracted from the different tissues were purchased from Ambion. The quality of the RNA was checked and found to be optimal, but information is not available on the precise region of a given organ that was actually sampled.
