# Supplementary material for: The protein tyrosine phosphatase receptor type R gene is an early and frequent target of silencing in human colorectal tumorigenesis
Source: Mol Cancer. 2009 Dec 16;8:124. doi: 10.1186/1476-4598-8-124 (PMC2801661; doi:10.1186/1476-4598-8-124)
Supplement: Additional file 3 — Supplementary Table 2. Primer sequences and PCR conditions used in this study. All primer sequences and PCR conditions used for quantitative RT-PCR, methylation analysis, and ChIP are reported. [file 1476-4598-8-124-S3.PDF]

Supplementary Table 2. Primer sequences and PCR conditions used in this study.

qRT-PCR

Gene	Primer set, forward/reverse	Lenght	Annealing temperature
PTPRR (transcript 1)	F:5'- CCAGCAGCAAATGTAATTGTG -3'<br>R:5'- GGAGACACAAACAGTTCAATACTG -3'	173 bp	53°
PTPRR (transcript 2)	F:5'- TTTTCTCAAGCTCTCATTTAACG -3'<br>R:5'- ATCTGTAAAGAATCATCAAACACG -3'	173 bp	55°
PBGD	F:5'- CAACGGCGGAAGAAAACAG -3'<br>R:5'- TCTCTCCAATCTTAGAGAGTG -3'	195 bp	55°

PTPRR-1 methylation analysis

Technique	Primer set, forward/reverse	Lenght	Annealing temperature
COBRA (fresh samples)	F:5'- GTTGGGTTTTTAGTAATTATAGTAAG -3<br>R:5'- CACCAACTTCAACCTCCCTA -3	368 bp	56°
COBRA (FFPE samples)	F:5'- GI*GATGGAGTTTATTTTGTTTTAAGT - 3<br>R:5'- CI*ACAAATACCTAACCTTCTAAAC -3	202 bp	52°
Bisulfite genomic sequencing	F:5'- AGGAATTCGTTGGGTTTTTAGTAATTATAGTAAG - 3<br>R:5'- AAGGATCCACCAACTTCAACCTCCCTA -3<br><br>I* = Inosine	383 bp	56°

ChIP

Gene / Locus	Primer set, forward/reverse	Lenght	Annealing temperature
RPL30	F:5'- CCGGATCCTAAAATTCCTGTC -3<br>R:5'- AGACATCTCTCCACGTGAATC -3	144 bp	58°
KCNA1	F:5'- TGACGGTGATGTCTGGGGAG -3<br>R:5'- ACGAGCGTGTTGGGGAACTG -3	187 bp	58°
SAT2	F:5'- ATCGAATGGAAATGAAAGGAGTCA -3<br>R:5'- GACCATTGGATGATTGCAGTCA -3	160 bp	58°
PTPRR (5' flanking transcript 1)	F:5'- GATGGAGCCTACTCTGCTCC -3<br>R:5'- TTCTGGACGCCCAGAAGCC -3	184 bp	60°
